# A Point Mutation at C151 of *Keap1* of Mice Abrogates NRF2 Signaling, Cytoprotection in Vitro, and Hepatoprotection in Vivo by Bardoxolone Methyl (CDDO-Me)[Fn fn5]

**DOI:** 10.1124/molpharm.123.000671

**Published:** 2023-08

**Authors:** Tonibelle Gatbonton-Schwager, Yoko Yagishita, Tanvi Joshi, Nobunao Wakabayashi, Harini Srinivasan, Takafumi Suzuki, Masayuki Yamamoto, Thomas W. Kensler

**Affiliations:** Translational Research Program (T.G.-S., Y.Y., T.J., N.W., T.W.K.) and Genomics & Bioinformatics (H.S.), Fred Hutchinson Cancer Center, Seattle, Washington and Department of Medical Biochemistry, Tohoku University Graduate School of Medicine, Sendai, Japan (T.S., M.Y.)

## Abstract

Bardoxolone methyl (CDDO-Me) is an oleanane triterpenoid in late-stage clinical development for the treatment of patients with diabetic kidney disease. Preclinical studies in rodents demonstrate the efficacy of triterpenoids against carcinogenesis and other diseases, including renal ischemia-reperfusion injury, hyperoxia-induced acute lung injury, and immune hepatitis. Genetic disruption of *Nrf2* abrogates protection by triterpenoids, suggesting that induction of the NRF2 pathway may drive this protection. Herein, we examined the effect of a point mutation (C151S) in KEAP1, a repressor of NRF2 signaling, at cysteine 151 in mouse embryo fibroblasts and mouse liver. Induction of target gene transcripts and enzyme activity by CDDO-Me was lost in C151S mutant fibroblasts compared with wild-type. Protection against menadione toxicity was also nullified in the mutant fibroblasts. In mouse liver, CDDO-Me evoked the nuclear translocation of NRF2, followed by increased transcript and activity levels of a prototypic target gene, *Nqo1*, in wild-type, but not C151S mutant, mice. To test the role of KEAP1 Cys151 in governing the broader pharmacodynamic action of CDDO-Me, wild-type and C151S mutant mice were challenged with concanavalin A to induce immune hepatitis. Strong protection was seen in wild-type but not C151S mutant mice. RNA-seq analysis of mouse liver from wild-type, C151S mutant, and *Nrf2*-knockout mice revealed a vigorous response of the NRF2 transcriptome in wild-type, but in neither C151S mutant nor *Nrf2*-knockout, mice. Activation of “off-target” pathways by CDDO were not observed. These data highlight the singular importance of the KEAP1 cysteine 151 sensor for activation of NRF2 signaling by CDDO-Me.

**SIGNIFICANCE STATEMENT:**

KEAP1 serves as a key sensor for induction of the cytoprotective signaling pathway driven by the transcription factor NRF2. Mutation of a single cysteine (C151) in KEAP1 abrogates the induction of NRF2 signaling and its downstream cytoprotective actions in vitro and in vivo by bardoxolone methyl (CDDO-Me), a drug in late-stage clinical development. Further, at these bioeffective concentrations/doses, activation of “off-target” pathways by CDDO-Me are not observed, highlighting the singular importance of NRF2 in its mode of action.

## Introduction

The transcription factor NF-E2-related factor 2 (NRF2) belongs to the Cap’n’Collar basic leucine zipper family, assembling into a complex with its heterodimeric partners, small Maf proteins, that bind to antioxidant response elements (AREs) to activate or repress a battery of NRF2 target genes. When *Nrf2*-knockout mice were first established, their phenotype showed diminished expression of xenobiotic detoxication enzymes, along with enhanced sensitivity to toxins (Itoh et al., 1997; Chan and Kan, 1999; Enomoto et al., 2001). Kelch-like ECH-associated protein 1 (KEAP1), a repressor facilitating the proteasomal degradation of NRF2, was identified thereafter as a critical factor affecting NRF2 fate and magnitude of toxin sensitivity (Itoh et al., 1999, Kobayashi et al., 2004). Based on these fundamental findings, NRF2 research accelerated to define NRF2 as a critical defense against electrophiles and reactive oxygen species (Kensler et al., 2007, Yamamoto et al., 2018). Comprehensive, high-throughput transcriptome profiling reveals an ever-broadening range of NRF2 target genes influencing electrophilic and oxidative stresses, anti-inflammatory responses, cell metabolism, cell proliferation/differentiation, and cell death pathways, involving ∼300 potential direct NRF2 target genes (Thimmulappa et al., 2002, Malhotra et al., 2010, Yamamoto et al., 2018, Yagishita et al., 2023). As a consequence of this broad, cellular prosurvival response, there has been a concerted effort to identify and use inducers of NRF2 signaling for disease prevention and treatment in preclinical models and now in clinical trials (Copple et al., 2017, Cuadrado et al., 2019, Dinkova-Kostova and Copple, 2023).

Prior to the recognition and characterization of the KEAP1-NRF2-ARE system, Talalay et al. (1988) deduced that many inducers contain, or acquire by metabolism, a distinctive chemical and structural feature, electrophilic olefin, or related electron-deficient centers that function as Michael reaction acceptors. They predicted the existence of a sensor molecule with highly reactive cysteines responding to a diverse chemical library of inducers. Upon isolating KEAP1 as a binding partner of NRF2, the Yamamoto group (Itoh et al., 1999) reported that KEAP1 possessed 25 and 27 cysteines in human and mouse, respectively. Several of these cysteines in KEAP1 are adjacent to basic residues and expected to have lower PKa values and consequent increased reactivity, consistent with the idea that KEAP1 was the direct sensor molecule.

Wakabayashi et al. (2004) initiated a molecular-genetic approach to probe the role of specific KEAP1 cysteines; absence of either C273 or C288 or both abrogated repressor activity in hepatoma cells. Zhang and Hannink (2003) demonstrated that C273 and C288 are required for KEAP1-dependent ubiquitination of NRF2. They also identified cysteine 151 (C151) as a target for the inducer sulforaphane, wherein binding enabled NRF2 to escape KEAP1-dependent degradation, leading to increased nuclear accumulation and transcriptional upregulation of cytoprotective genes. Mass spectrometry has also been used to identify KEAP1 cysteines modified by several inducers (Dinkova-Kostova et al., 2002; Hong et al., 2005; Luo et al., 2007).

As reviewed in Yamamoto et al. (2018), a cysteine code defining responses for different classes of inducers was identified. Macrophages harboring a C151S mutant did not respond to a large group of electrophiles (Takaya et al., 2012). C288 was favored for sensing the endogenous inducer PGJ2,15-deoxy-Δ12,14-prostaglandin J2 (15*Δ*-PGJ_2_) (Saito et al., 2015). Although a comprehensive cataloging of all cysteine targets for most inducers has not been undertaken, an 11-cysteine-less mutant cell line was insensitive to most electrophilic inducers but retained basal repressor activity (Suzuki et al., 2019).

Oleanane triterpenoids, developed by Sporn, Honda, and Gribble, are exceedingly potent inducers with efficacy in cell culture at nanomolar concentrations (Dinkova-Kostova et al., 2005). Two triterpenoids, 1-(2-cyano-3,12,28-trioxooleana-1,9(11)-dien-28-yl)-1H-imidazole (CDDO-Im) and methyl-2-cyano-3,12-dioxooleano-1,9-dien-28-oate (bardoxolone methyl; CDDO-Me), have been widely studied in preclinical models for efficacy against carcinogenesis and other diseases. CDDO-Im, unlike any other chemopreventive tested, completely abrogated the hepatocarcinogenicity of aflatoxin B_1_ in a lifetime bioassay in rats (Johnson et al., 2014), acute manifestations of hepatotoxicity elicited by acetaminophen (Reisman et al., 2009), and concanavalin A (ConA) (Osburn et al., 2008) as well as hyperoxia-induced acute lung injury (Reddy et al., 2009) and renal ischemia-reperfusion injury (Liu et al., 2014) in mice. CDDO-Im has been described previously as a C151-preferring inducer (Saito et al., 2015), although other cysteines in KEAP1 are likely to be functional targets as well (Meng et al., 2020). Unlike other triterpenoids, CDDO-Me has undergone substantial clinical development (Kanda and Yamawaki, 2020).

In this study, we sought to address the role of KEAP1 C151 as a critical target for the cytoprotective actions of CDDO-Me. Unexpectedly, it appears this single cysteine exclusively determines, at low concentrations, the responsiveness of the KEAP1-NRF2-ARE pathway to the enzyme-inducing and cell survival responses in vitro and in vivo. Further, at these concentrations, activation of “off-target” pathways by CDDO-Me are not observed.

## Materials and Methods

### Materials

CDDO-Me was purchased from Toronto Research Chemicals (North York, ON). CDDO-Im was purchased from Tocris (Bristol, United Kingdom). Bovine serum albumin, Tween-20, flavin adenine dinucleotide, 3,3′-methylene-bis (dicoumarol), D-glucose 6-phosphate sodium salt, glucose-6-phosphate dehydrogenase, thiazolyl blue tetrazolium bromide (MTT), and ConA were purchased from Sigma (St. Louis, MO). 2-Methyl-1,4-naphthoquinone (menadione) and digitonin were purchased from Acros (Antwerp, Belgium), 15Δ-PGJ_2_ from Cayman Chemical (Ann Arbor, MI), and CdCl_2_ from Thomas Scientific (Swedesboro, NJ). Tris, NADP, and EDTA were purchased from BioRad (Hercules, CA), Roche (Indianapolis, IN), and Thermo Fisher Scientific (Waltham, MA), respectively.

### Cell Culture

Stable male wild-type and *Keap1*^C151S^ (C151S) murine embryonic fibroblast (MEF) cell lines were established from *Keap1*^C151S^ mice created by the Yamamoto laboratory (Tohoku University, Japan) (Saito et al., 2015) as described previously (Wakabayashi et al., 2010). The cell lines were cultured in Iscove’s modified Dulbecco’s medium (Gibco, Billings, MT) containing L-glutamine and 25 mM Hepes and supplemented with 10% FBS (Gibco) and 100 µg/mL Primocin (Invivogen, San Diego, CA) in a 37°C humidified incubator with an atmosphere of 5% CO_2_.

### RNA Preparations from Cells and Analysis of *Nqo1* mRNA Expression

MEFs were plated at 4 × 10^4^ – 5 × 10^4^ cells per well in six-well plates for 24 hours followed by treatment with CDDO-Im (1, 3, 10, 30 nM), CDDO-Me (1, 3, 10, 30 nM), 15Δ-PGJ_2_ (10 µM), CdCl_2_ (30 µM), or vehicle control for an additional 24 hours to reach 70%–80% cell confluency at time of harvest. RNA was extracted using TRIzol Reagent (Thermo Fisher Scientific), and cDNA was synthesized from 100 ng of RNA with qScript cDNA Synthesis Kit (Quant Biosciences, Beverly, MA). Maxima SYBR Green/ROX qPCR Master Mix (Thermo Fisher Scientific) and QuantStudio 7 (Applied Biosystems, Waltham, MA) were used for quantitative real-time polymerase chain reaction (qPCR).

### NQO1 Enzyme Activity

MEFs were plated at 5 × 10^3^ – 8 × 10^3^ cells per well in 48-well plates for 24 hours followed by treatment with CDDO-Im (1 and 30 nM), CDDO-Me (1 and 30 nM), or DMSO control for 48 hours to reach 90%–95% cell confluency at the time of analysis. Cells were lysed with 0.08% digitonin/EDTA (2 mM, pH 7.8) for 20 minutes at 37°C. The assay was performed as described previously (Prochaska and Santamaria, 1988). Briefly, 80 µL of cell lysate was incubated at room temperature for 5 minutes with 200 µL of the reaction mixture: NAD(P)H:quinone dehydrogenase 1 (NQO1) assay buffer (25 mM Tris, pH 7.4, 0.66 mg/mL bovine serum albumin, 0.01% Tween-20) mixed with cofactors (5 µM flavin adenine dinucleotide, 1 mM G6P, 30 µM NADP, 30 U G6P, 0.3 mg/mL MTT) and 50 µM menadione. For mouse liver samples, approximately 100 mg of livers were lysed and used for the assay. Spectrophotometric measurement was performed using SpectraMax M5 plate reader (Molecular Devices). Protein concentration was measured using the BCA reagent (Thermo Fisher Scientific), which was used for the normalization of enzyme activity.

### Menadione Assay

MEFs were plated at 2 × 10^3^ cells per well in 96-well plates for 24 hours followed by treatment with CDDO-Im (30 nM), CDDO-Me (30 nM), or DMSO as vehicle control. After 24 hours, MEFs were treated with menadione at the indicated concentrations along with CDDO-Im (30 nM), CDDO-Me (30 nM), or DMSO for an additional 24 hours. Cell viability was examined by MTT assay as described previously (Hoare and Hughes, 2004).

### Mice

*Nrf2*-knockout mice (Itoh et al., 1997) and C151S knock-in mice (C151S) (Saito et al., 2015) were generated by the Yamamoto laboratory (Tohoku University, Japan). C151S mice were developed by CRISPR-Cas9 genome editing technology as described previously (Saito et al., 2015). Wild-type mice [C57BL/6J (B6(Cg)-Tyr^c-2J^/J)] were obtained from the Jackson Laboratory. All mice used in the experiments were albino C57BL/6J background [B6 (Cg)-Tyr^c-2J^/J], male, and 7–10 weeks old. Homozygous C151S mice and *Nrf2*-knockout mice were used for mating and for experiments. The C151S allele was genotyped by TaqMan qPCR methods using purified mouse tail DNA. All animal experiments were approved by the Institutional Animal Care and Use Committee at the Fred Hutchinson Cancer Center.

### Mouse Treatment with the NRF2 Inducers

Mice were orally administered CDDO-Me [30 μmol/kg body weight (BW)], CDDO-Im (30 μmol/kg BW), or vehicle (10% DMSO, 10% Cremophor-EL, and PBS) alone. According to the experimental design, single or repeated treatments (every other day, a total of three doses) were performed. At 24 hours after the last treatment, tissues were collected for further analysis.

### Acute Immune Hepatitis Model

Mice were pretreated with a total of three doses of CDDO-Me (30 μmol/kg BW), CDDO-Im (30 μmol/kg BW), or vehicle alone. At 24 hours after the last treatment, mice were administrated ConA (12 mg/kg BW) by intravenous injection; 8 hours later, serum was collected for analyses. To assess the extent of hepatotoxicity, alanine aminotransferase (ALT) activity in serum was measured using an assay kit from APExBIO (Houston, TX). For histologic analysis, livers were immersed in 4% paraformaldehyde, and H&E-stained sections were prepared. Imaging was performed using a Nikon Eclipse E800 microscope and a Zeiss AxioCam MRc camera.

### Protein Preparation and Immunoblot Analysis

Mice were treated with CDDO-Me (30 μmol/kg BW) or vehicle (10% DMSO, 10% Cremophor-EL, and PBS) alone. Three hours after treatment, livers were collected to examine the nuclear translocation of NRF2. Nuclear extracts were prepared using NE-PER Nuclear and Cytoplasmic Extraction Regents (Thermo Scientific) according to the manufacturer’s directions. The protein samples were subjected to 8% SDS-PAGE and transferred to a polyvinylidene difluoride membrane. Specific protein signals were detected by anti-NRF2 (sc-13032, Santa Cruz Biotechnology Dallas, TX) and anti–*α*-tubulin (MAB1864, Millipore, Burlington, MA) antibodies. Image J (National Institutes of Health) was used for quantification analyses.

### RNA Preparation from the Livers and qPCR Analysis

The harvested livers were homogenized in Trizol (Thermo Fisher Scientific), and total RNA was extracted, followed by cleanup using an RNeasy Mini Kit (Qiagen). cDNA was synthesized using the qScript system (Quanta Biosciences), and qPCR was performed by using Maxima SYBR Green/ROX qPCR Master Mix (Thermo Fisher Scientific) and QuantStudio 7 (Applied Biosystems). The primers are shown in Supplemental Table 1. Expression levels of each gene were normalized to *Actb* and calculated relative to the control.

### Library Construction and RNA Sequencing

Total RNA derived from the livers of wild-type, C151S, and *Nrf2*-knockout mice treated with vehicle (*n* = 5) and CDDO-Me (*n* = 5) in each genotype were used. RNA quality check, library construction, and sequencing were performed at the Fred Hutchinson Cancer Center Shared Resources Cores for Genomics and Bioinformatics. Total RNA integrity was affirmed and quantified using an Agilent 4200 TapeStation (Agilent Technologies, Inc., Santa Clara, CA) and Trinean DropSense96 spectrophotometer (Caliper Life Sciences, Hopkinton, MA), respectively. RNA-seq libraries were prepared from total RNA (TruSeq Stranded mRNA kit, Illumina, Inc., San Diego, CA), and library size distribution was validated (Agilent 4200 TapeStation, Agilent Technologies, Santa Clara, CA). Additional quality control of the library, combining of pooled indexed libraries, and cluster optimization was performed using Life Technologies’ Invitrogen Qubit 2.0 Fluorometer (Life Technologies-Invitrogen, Carlsbad, CA). RNA-seq libraries were pooled (30-plex) and clustered onto a P3 flow cell. Samples were sequenced using an Illumina NextSEq 2000 using a paired-end, 50 base read-length strategy. Raw data have been deposited in Gene Expression Omnibus; the accession number is GSE222256.

### Bioinformatics Analysis

Quality assessment of the raw sequencing data, in Fastq format, was performed with fastp v0.20.0 (Chen et al., 2018) to ensure that data had high base call quality, expected guanine-cytosine content for RNA-seq, and no overrepresented contaminating sequences. No reads or individual bases were removed during this assessment step. The fastq files were aligned to the UCSC mouse mm10 reference assembly using STAR v2.7.7 (Dobin et al., 2013). STAR was run with the parameter “–quantMode GeneCounts” to produce a table of raw gene-level counts with respect to annotations from mouse GENCODE build M23. A full list of alignment parameters is provided in Supplemental Methods. To account for stranded library preparation, only counts from the second strand were retained for further analysis. The quality of the alignments was evaluated using RSeQC v3.0.0 (Wang et al., 2012), including assessment of bam statistics, read-pair inner distance, and read distribution. Differential expression analysis was performed with edgeR v3.36.0 (Robinson et al., 2010) to identify the differences between mice treated with CDDO-Me and vehicle of wild-type, C151S, and *Nrf2*-knockout with five biologic replicates in each group. Genes with very low expression across all samples were flagged for removal by filterbyExpr, and trimmed mean of M values normalization was applied with calcNormFactors to account for differences in library composition and sequencing depth. We constructed a design matrix to incorporate potential batch effects related to date of sample isolation, after which the dispersion of expression values was estimated using estimateDisp. Testing for each gene was then performed with the QL F-test framework using glmQLFTest, which outputs for each gene a *P* value, a log_2_(fold change) value, and a Benjamini-Hochberg corrected false discovery rate (FDR) to control for multiple testing. The results were plotted using ggplot2 v3.3.5 (Wickham 2016). To investigate pathways and gene signatures, preranked gene set enrichment analysis was performed using clusterProfiler v4.2.2 (Yu et al., 2012). Genes were ranked by -log_2_(FDR) multiplied by sign of the corresponding log fold change (FC) value, to take account of both significance and direction of change, and gene signatures from the Kyoto Encyclopedia of Genes and Genomes and Gene Ontology databases were assessed for enrichment of differentially expressed genes.

### Statistical Analysis

GraphPad Prism 9 software was used for statistical analysis of data sets. The nonlinear regression package was used to determine TC_50_ values. For the comparison of two groups, unpaired two-tailed Student’s *t* test was used; for more than two groups, one-way ANOVA was used followed by Dunnett’s or Tukey’s test as described in the relevant legends.

## Results

### CDDO-Me Induces Gene Expression and Enzyme Activity of NQO1 in a KEAP1 C151–Dependent Manner in MEFs

**Fig. 1.**
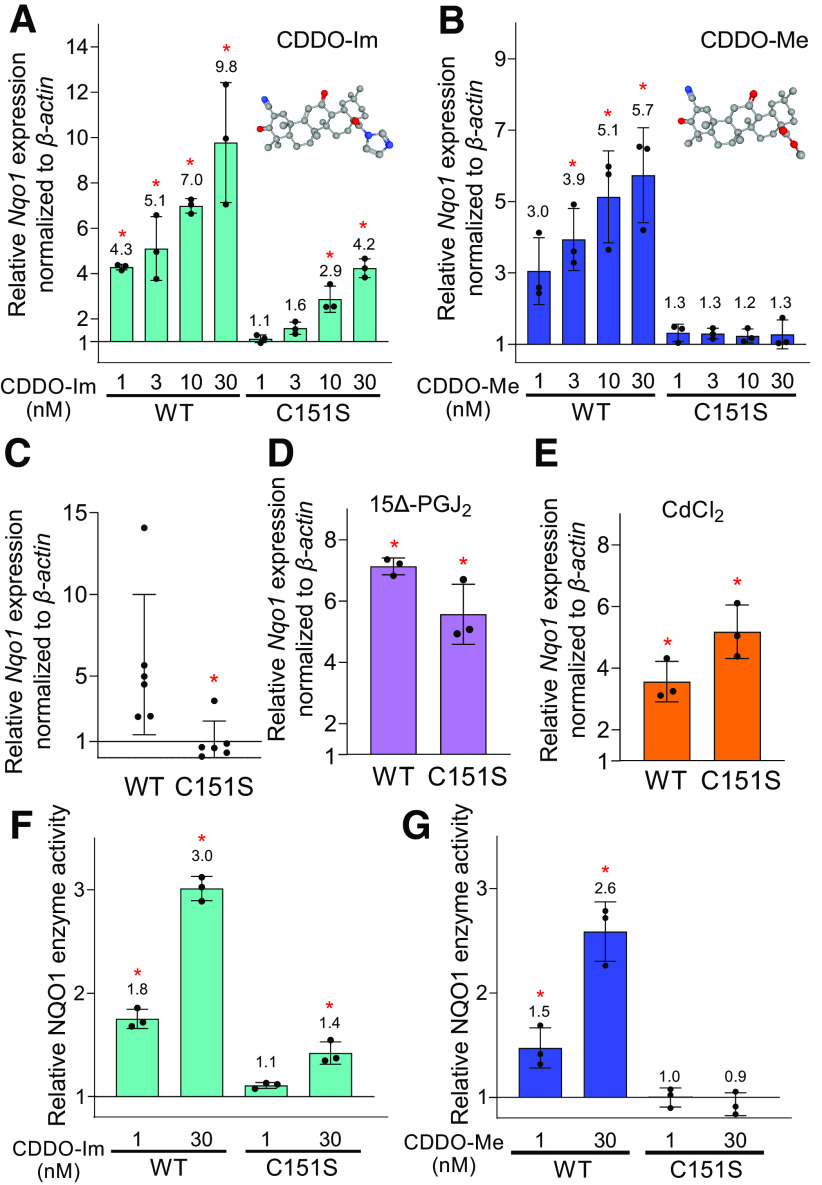
CDDO-Me induces mRNA expression and enzyme activity of NQO1 in a KEAP1 cysteine 151 -dependent manner in MEFs. (A and B) *Nqo1* mRNA expression in wild-type (WT) and C151S MEFs treated by CDDO-Im (A) or CDDO-Me (B). The MEFs were treated for 24 hours with the indicated concentrations of the two compounds. The chemical structures of CDDO-Im and CDDO-Me are indicated in the top corner of (A) and (B), respectively. The expression levels in the vehicle control of WT or C151S were set as 1. *P < 0.05 using one-way ANOVA. (C) *Nqo1* mRNA expression in WT and C151S MEFs under homeostatic conditions. The MEFs cultured without any treatment were used to determine the basal level of *Nqo1*. The expression level in C151S MEFs was set as 1. *P < 0.05 using Student’s *t* test. (D and E) *Nqo1* mRNA expression in WT and C151S MEFs treated with 10 µM 15Δ-PGJ_2_ (D) or 30 µM CdCl_2_ (E) for 24 hours. The expression levels in the vehicle control in each genotype of MEFs were set as 1. *P < 0.05 using Student’s *t* test. Gene expression was normalized by *β-actin* for all mRNA data. (F and G) NQO1 enzyme activity in WT and C151S MEFs treated with CDDO-Im (F) or CDDO-Me (G). Enzyme activities of the vehicle control in each genotype of MEFs were set as 1. *P < 0.05 using one-way ANOVA followed by Dunnett’s test. Three independent experiments were performed for each assay. Data are presented as means ± S.D.

To examine the potential critical contribution of KEAP1 C151 to triterpenoid-driven activation of NRF2, MEFs derived from *Keap1 C151S* knock-in mice (Saito et al., 2015) were used. In C151S mice, a cysteine is replaced with serine at position 151 of the *Keap1* sequence. Using *Nqo1* expression as a marker of Nrf2 activation, the responses to CDDO-Im and CDDO-Me were measured. As shown in Fig. 1, A and B, both CDDO-Im and CDDO-Me elevated *Nqo1* mRNA expression in dose-dependent manners in wild-type MEFs. Although 1 and 3 nM of CDDO-Im did not show induction of *Nqo1* expression in C151S MEFs, higher concentration of CDDO-Im (i.e., 10 and 30 nM) demonstrated higher levels (2.9- and 2.4-fold, *P* < 0.05) of *Nqo1* transcripts compared with vehicle-treated MEFs (Fig. 1A). Notably, *Nqo1* expression did not increase in C151S MEFs with any concentrations of CDDO-Me (Fig. 1B). In wild-type MEFs, CDDO-Im and CDDO-Me showed equivalent potency in the induction of *Nqo1*; however, higher efficacy at the dose of 30 nM was observed with CDDO-Im as compared with CDDO-Me. Although *Nqo1* is a prototypical NRF2 target gene, the responses of two additional target genes, carbonyl reductase 3 (*Cbr3*) and glutathione *S*-transferase M1 (*Gstm1*), were measured to impute generalization of the interactions between genotype and triterpenoid. Dose-dependent increases in transcript levels were seen for both genes with CDDO-Im and CDDO-Me in wild-type mice; no appreciable induction was observed with either agent in the C151S mutant mice (data not shown).

It was also shown that the basal level of *Nqo1* expression in nontreated C151S MEFs was notably lower than that in wild-type MEFs (Fig. 1C), an observation consistent with previous data in transgenic mice expressing *Keap1 C151S* in a *Keap1*-null background (Yamamoto et al., 2008). In addition to Cys151, Cys273, and Cys288 located in the intervening region are well-recognized sensor cysteines in KEAP1. It has been reported that 15Δ-PGJ_2_ utilizes specific preference for Cys288, whereas cadmium chloride (CdCl_2_) harbors dependency on Cys226/613/622/624 of KEAP1 (but is independent of Cys151/273/288) (Saito et al., 2015). Indeed, the treatments with 15Δ-PGJ_2_ and CdCl_2_ exhibited comparative levels of induction in *Nqo1* expression in wild-type and C151S MEFs (Fig. 1, D and E), thereby confirming the residual function of KEAP1 harboring a point mutation on C151 with other classes of inducers. The response of NRF2 to CDDO-Im and CDDO-Me was further investigated by measuring NQO1 enzyme activity in MEFs. Wild-type MEFs showed substantial elevation of NQO1 enzyme activity by treatment with CDDO-Im and CDDO-Me (1 and 30 nM), and C151S MEFs showed elevated NQO1 activity only with 30 nM, but not 1 nM, CDDO-Im (Fig. 1, F and G). Of note, C151-dependent nuclear accumulation of NRF2 protein has been reported previously with CDDO-Im in MEFs (Saito et al., 2015). Importantly, CDDO-Me did not show increased NQO1 activity with either 1 or 30 nM, in agreement with the mRNA expression levels of *Nqo1* (Fig. 1B). The basal level of NQO1 enzyme activity (the ratio of wild-type to C151S is 1.0:0.27) was lower in C151S than wild type (*P* = 0.025). Together, these data demonstrate that KEAP1 C151 serves as an important sensor of triterpenoid analogs with full dependency of CDDO-Me on C151 and a strong but incomplete dependency for CDDO-Im. Using an epoxide-containing analog of CDDO-Me, Wong et al. (2016) previously characterized irreversible modification of multiple cysteines (257/273/288/434/489/613) in recombinant mouse KEAP1, wherein form does not appear to match function.

### CDDO-Me Ameliorated Menadione-Induced Cytotoxicity in Wild-Type MEFs but not in C151S MEFs

**Fig. 2.**
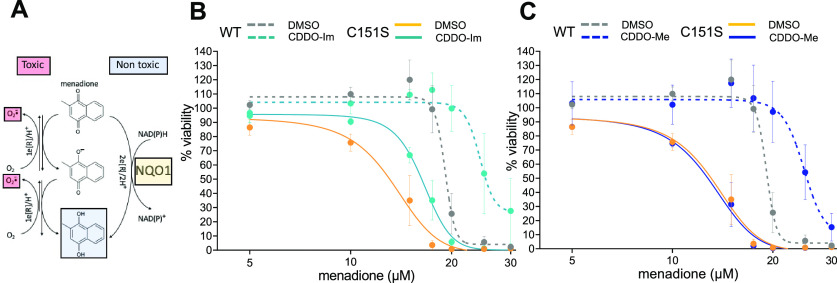
CDDO-Me ameliorates menadione-induced cytotoxicity in wild-type (WT), but not in C151S, MEFs. (A) Menadione is metabolized with a production of superoxide and hydrogen peroxide, leading to oxidative and nitrosative damage. NQO1 detoxifies the menadione quinone by reducing it to hydroquinone, which undergoes a subsequent detoxification process. (B and C) WT and C151S MEFs were treated with CDDO-Im (B) or CDDO-Me (C) for 24 hours, followed by an administration of menadione with indicated concentrations (0–30 µM). Cell viability after 24 hours of menadione treatment was determined. Three independent experiments were performed. Data in the graphs are presented as the mean ± S.E. of the means of each concentration, genotype, and triterpenoid. Each individual experiment included three technical replicates at each concentration. Concentration-response curves were fit using GraphPad Prism; TC_50_ values were calculated from each individual experiment: means ± S.D. from the three experiments are presented in Table 2.

To investigate the role of KEAP1 C151 on a NRF2-mediated cytoprotective response evoked by the triterpenoids, wild-type and C151S MEFs were challenged with menadione. Menadione is metabolized with a production of superoxide and hydrogen peroxide, leading to oxidative damage, wherein NQO1 detoxifies quinones by catalyzing an obligatory two-electron reduction to bypass generation of semiquinone radicals and reactive oxygen species via redox cycling reactions (Fig. 2A) (Ross and Siegel, 2021). As shown in Fig. 2B, CDDO-Im showed a protective effect against menadione-mediated toxicity in wild-type MEFs, exhibiting a TC_50_ value of 24.2 µM, which was higher than vehicle control (TC_50_ of 18.8 µM) (Table 1). Although CDDO-Im demonstrated a mildly shifted dose-response curve as compared with vehicle in C151S MEFs (Fig. 2B), protection was not statistically significant (Table 1; TC_50_ of 13.8 µM in vehicle versus 16.7 µM in CDDO-Im). The mild cytoprotection observed in C151S MEFs treated by CDDO-Im (30 nM) may be, at least partially, attributable to elevated *Nqo1* expression induced by the higher concentrations of CDDO-Im in C151S MEFs (Fig. 1, A and F). Importantly, CDDO-Me notably suppressed menadione-induced cytotoxicity in wild-type MEFs, whereas its protection was completely abrogated in C151S MEFs (Fig. 2C; Table 1). A comparison of TC_50_ values between vehicle-treated wild-type and C151S MEFs indicated a difference in the sensitivity for menadione-induced cytotoxicity (*P* = 0.035). It is plausible that the lower mRNA expression and enzyme activity of NQO1 shown in C151S than wild-type MEFs at the basal level (Fig. 1C) contribute to KEAP1 C151S–dependent differences in menadione sensitivity. In summary, these data demonstrate that NQO1-mediated cytoprotective effects induced by the triterpenoids occur in a KEAP1 Cys151–dependent manner in vitro, wherein CDDO-Me shows higher specificity than CDDO-Im regarding the reliance on KEAP1 C151.

**TABLE 1:** TC_50_ values for menadione cytotoxicity observed in wild-type and C151S MEFs treated with CDDO-Im or CDDO-Me Concentrations of menadione, which reduce cell viability by 50% (TC_50_), were determined in wild-type and C151S MEFs based on the concentration-response curves. Data are presented as means ± S.D. obtained from three independent assays. One-way ANOVA followed by Dunnett’s test was performed.

Wild-Type MEFs	TC_50_ Menadione	S.D.	*P* Value
DMSO	18.8	± 1.06 µM	
CDDO-Im 30 nM	24.2	± 1.47 µM	0.003
CDDO-Me 30 nM	25.4	± 0.91 µM	0.001
			
C151S MEFs	TC_50_ Menadione	S.D.	*P* Value
DMSO	13.8	± 2.55 µM	
CDDO-Im 30 nM	16.7	± 0.71 µM	0.291 (n.s.)
CDDO-Me 30 nM	13.7	± 1.37 µM	0.998 (n.s.)

n.s., not significant.

### C151 of KEAP1 is Required for CDDO-Me–Induced Stimulation of NRF2 Signaling in Vivo

**Fig. 3.**
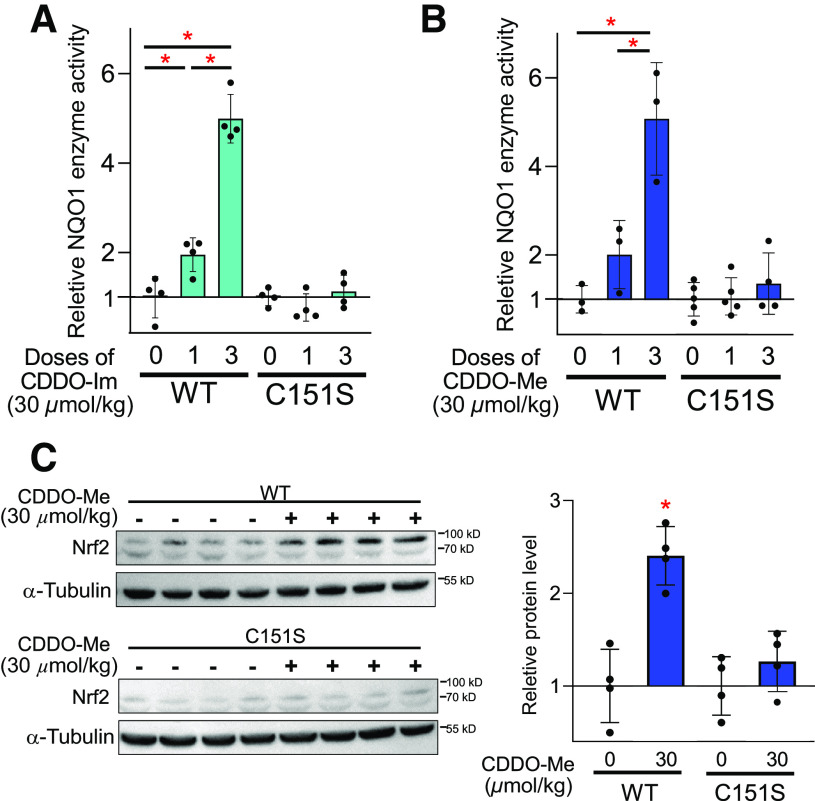
CDDO-Me does not induce activation of NRF2 in the livers of C151S mice. NQO1 enzyme activity in the livers of wild-type (WT) and C151S mice treated with single or repeated doses (a total of three doses) of CDDO-Im (A) and CDDO-Me (B) (*n* = 3–5). Enzyme activity in the vehicle-treated group was set as 1. The ratio of basal NQO1 activities in wild-type and C151S livers is 1.0:0.22). *P < 0.05 using one-way ANOVA. The data obtained from the different genotypes were not directly compared by statistical analysis. (C) Immunoblot analyses of NRF2 and *α*-tubulin in the nucleus extracts derived from the livers of WT and C151S mice treated with one dose of CDDO-Me (*n* = 4). After 3 hours of treatment, the livers were harvested for analysis. The quantification of band intensity is shown in the right panel. *P < 0.05 using Student’s *t* test. Data are presented as means ± S.D.

Wild-type and KEAP1 C151S mice were treated with either a single dose or repeated doses (a total of three doses) of CDDO-Im or CDDO-Me, and NQO1 enzyme activity in the livers was examined. Both single and repeated doses of CDDO-Im showed higher activity (2.0- and 5.0-fold, *P <* 0.05) of hepatic NQO1 as compared with the respective vehicle controls; no induction of NQO1 activity was observed in the livers of C151S mice (Fig. 3A). Similarly, CDDO-Me, particularly following repeated doses, elicited induction of NQO1 activity (2.0- and 5.1-fold, *P* < 0.05) in the livers of wild-type mice, which was abolished in C151S mice (Fig. 3B). CDDO-Im and CDDO-Me showed nearly identical induction of NQO1 activity in vitro (Fig. 1, A and B) and in the livers of wild-type mice (Fig. 3, A and B). Activation of NRF2 signaling by CDDO-Me was verified by examining nuclear accumulation of NRF2 in the livers. A 2.5-fold increase in NRF2 accumulation was detected in wild-type mice 3 hours after administration of one dose of CDDO-Me (30 µmol/kg); no difference in NRF2 protein accumulation was detected in the nucleus between vehicle and CDDO-Me of C151S mice (Fig. 3C). These data demonstrate that Cys151 of KEAP1 has a critical contribution to the induction of NRF2 signaling elicited by CDDO-Me in vivo.

### A Point Mutation at C151 of KEAP1 Abrogates CDDO-Me–Mediated Hepatic Protection in a Concanavalin A Model of Acute Hepatitis in Mice

**Fig. 4.**
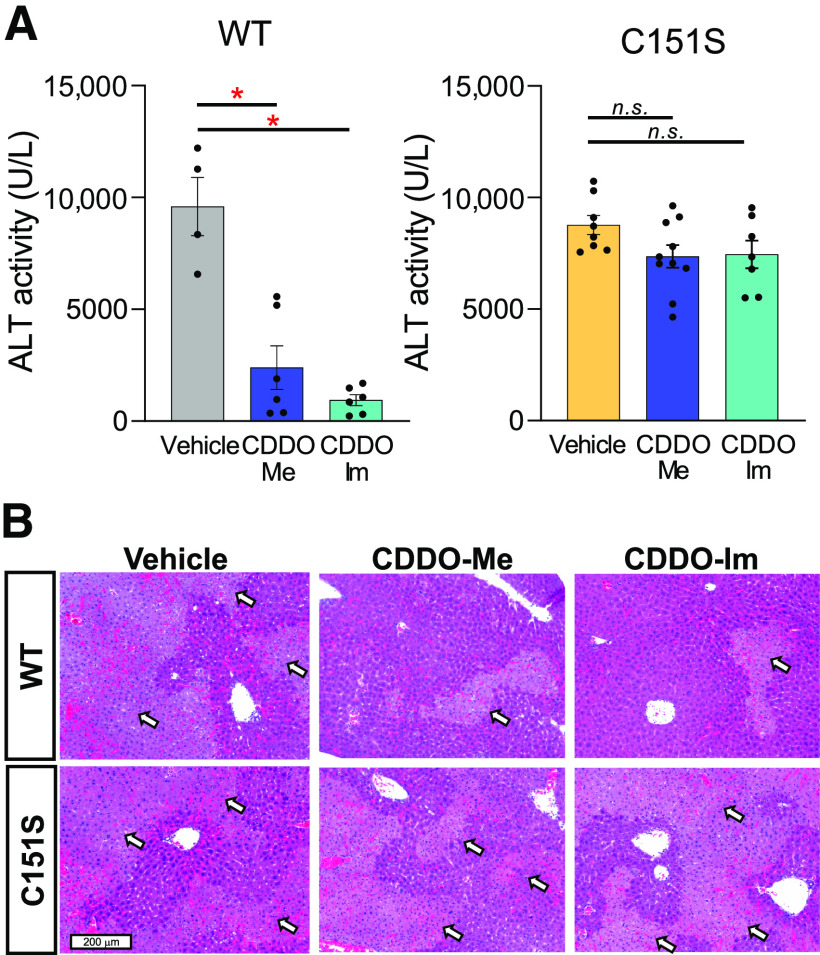
Point mutation at C151 of KEAP1 abrogates CDDO-Me–mediated hepatic protection in a ConA model of acute hepatitis in mice. After 8 hours of ConA challenge, serum samples were collected and ALT levels were determined in the mice pretreated with vehicle, CDDO-Me, or CDDO-Im (a total of three doses) (*n* = 4 – 10). *P < 0.05 using one-way ANOVA followed by Tukey’s test. Scale bar, 200 μm. n.s., not significant; WT, wildtype.

The contribution of cysteine sensor C151S in KEAP1 to cytoprotective functions induced by CDDO-Me was further studied in vivo using a model of ConA-induced acute immune hepatitis. Mice were pretreated with CDDO-Im or CDDO-Me (a total of three doses), followed by ConA administration. Liver damage was assessed by serum transaminase activities (ALT) in wild-type and C151S mice 8 hours later. ConA administration evoked high ALT levels in vehicle-treated wild-type mice, which was notably suppressed by CDDO-Im and CDDO-Me (Fig. 4A). ALT was similarly high in the vehicle-treated C151S mice, which was not abrogated by CDDO-Im and CDDO-Me pretreatment. Microscopic examination of H&E-stained liver sections revealed severe and extensive necrosis in ConA-challenged, vehicle-treated, wild-type, and C151S mice. CDDO-Me and CDDO-Im afforded substantial, albeit incomplete, protection against this necrosis in wild-type, but not C151S, mice (Fig. 4B), in accord with the effects on serum ALT levels. These data demonstrated that KEAP1 C151 was required to induce triterpenoid-mediated protection against acute hepatitis in mice.

### RNA-Seq Analysis Defines an Exclusive NRF2 Signature for CDDO-Me–Induced Genes in Mouse Liver with a Total Dependency upon C151 of KEAP1

**Fig. 5.**
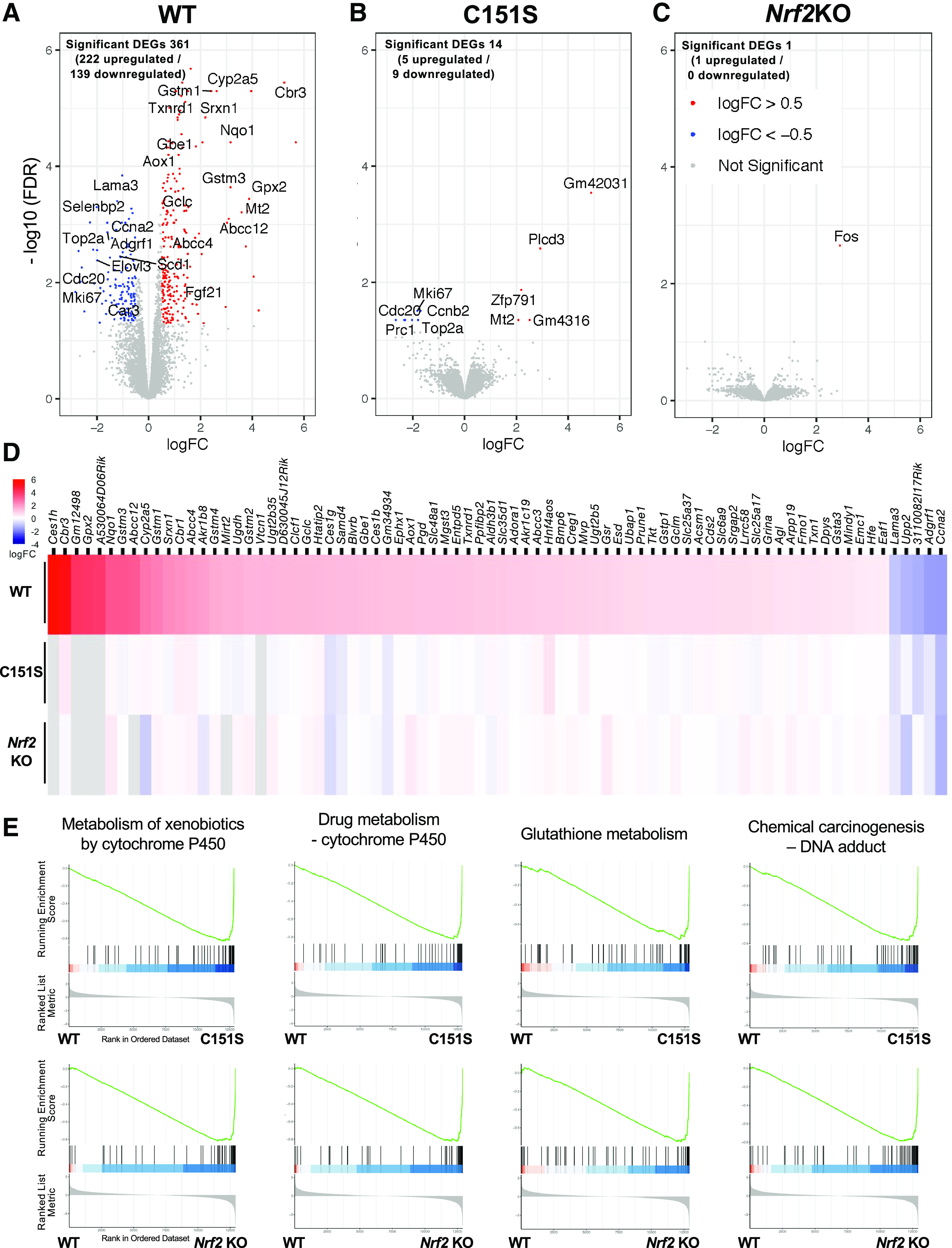
A point mutation at C151 of KEAP1 abrogates the global expression profile of Nrf2-dependent genes induced by CDDO-Me in mouse liver. (A–C) Volcano plot showing DEGs in the livers of wild-type (WT) (A), C151S (B), and *Nrf2*-knockout (KO) (C) mice. DEGs were determined by comparative analysis between vehicle-treated and CDDO-Me–treated mice (CDDO-Me 30 µmol/kg, single dose, oral, *n* = 5 for each treatment/genotype). Significance was determined by requiring Benjamini-Hochberg controlled false discovery rate of 5% (FDR <0.05) and a fold change of at least 1.4 (|log_2_FC| > 0.5). Red dots represent statistically significantly upregulated DEGs, blue dots represent downregulated DEGs, and gray dots represent genes with unchanged expression. (D) Heatmap illustrating DEGs in wild-type, C151S, and *Nrf2*KO mice. Listed genes are NRF2-dependent DEGs induced by CDDO-Me in wild-type mice, which were determined by screening the common DEGs between WT-vehicle versus WT-CDDO-Me and WT-CDDO-Me versus *Nrf2*KO-CDDO-Me. Cutoff of FDR <0.01 was applied, resulting in 78 genes shown in the list. Gray color indicates a nondetectable expression level. (E) Enrichment plots (Kyoto Encyclopedia of Genes and Genomes) comparing WT-CDDO-Me versus C151S-CDDO-Me and WT-CDDO-Me versus *Nrf2*KO-CDDO-Me are shown with the gene set classifications of “Metabolism of xenobiotics by cytochrome P450”, “Drug metabolism – cytochrome P450”, “Glutathione metabolism”, “Chemical carcinogenesis – DNA adduct” pathways.

RNA-seq was performed in the livers of the mice treated with one dose of CDDO-Me (30 µmol/kg) in wild-type, C151S, and *Nrf2*-knockout mice. In wild-type mice, 361 genes were shown to be statistically significantly differentially expressed genes (DEGs) between vehicle and CDDO-Me treatment (filtering criteria: FDR <0.05, absolute logFC ≥0.5), of which 222 were upregulated DEGs including well known NRF2 target genes such as *Nqo1*, *Gstm1*, *Gpx2*, and *Cbr3*, and 139 downregulated DEGs including genes reported as NRF2 targets or genes associated with NRF2 signaling, such as *Lama3*, *Selebp2*, *Ccna2*, and *Elovl3* (Fig. 5A). In C151S mice, only 14 genes were identified as DEGs between vehicle and CDDO-Me, of which five were upregulated, and nine were downregulated DEGs (Fig. 5B). In *Nrf2*-knockout mice, *Fos* was the only gene indicated as an upregulated DEG but was not identified as such in wild-type and C151S mouse liver (Fig. 5C). Thus, all of the DEGs induced or repressed by CDDO-Me in wild-type mice appear to be regulated in a *Nrf2*-dependent matter as little to no change in their expression levels were observed following treatment of C151S or *Nrf2*-knockout mice with CDDO-Me.

To narrow down the global gene expression profile, the common DEGs observed between vehicle-treated wild-type mice and CDDO-Me-treated wild-type mice and CDDO-Me–treated wild-type mice and CDDO-Me–treated *Nrf2*-knockout mice were screened and determined as CDDO-Me–induced, NRF2-dependent DEGs. As shown in Fig. 5D (FDR <0.01), C151S shares few if any of the most robust DEGs with wild-type after CDDO-Me treatment. C151S and *Nrf2*-knockout mice showed remarkably similar gene expression profiles. A cluster analysis was performed by targeting a larger number of genes determined as CDDO-Me–induced, NRF2-dependent DEGs (a total of 350 genes; FDR <0.05, logFC ≥0.5), verifying the marked similarity of profile in inducible gene expression between C151S and *Nrf2*-knockout, which was distinct from wild type (data not shown). Subsequently, qPCR analysis was performed to validate key DEGs identified by RNA-seq. To examine dose-dependent effects on the expression of these genes, the livers from mice treated with repeated doses of CDDO-Me (a total of three doses) were examined together with single-dosed mouse livers, as were used for RNA-seq. Herein, well known NRF2 target genes (i.e., *Nqo1*, *Gstm1*, *Gpx2*, *Srxn1*, *Crb3*), which were also identified as DEGs in wild-type mice, were examined. In wild-type mice, all genes showed higher expression in liver from CDDO-Me–treated as compared with vehicle-treated mice [3.5- (*Gstm1*) to 43.7-fold (*Crb3*) with single dose, all *P* < 0.05]. However, after Bonferroni correction for 30 multiple comparisons, only Nqo1 (one and three doses) was statistically significant (*P* < 0.00167). The induction level of *Nqo1*, *Gstm1*, and *Srxn1* by CDDO-Me was comparable between single and repeated doses, whereas *Gpx2* and *Crb3* showed a higher increase in the mice treated with repeated doses than a single dose (Table 2). In C151S and *Nrf2*-knockout mice, both single and repeated doses of CDDO-Me did not show induction of gene expression in examined genes. Although *Cbr3* showed an exceedingly modest (less than twofold, *P* < 0.05) elevation in its expression in *Nrf2*-knockout when repeated doses of CDDO-Me were administrated, the induced expression of *Cbr3* in *Nrf2*-knockout was approximately half of its basal expression in wild-type. In C151S mice, all examined genes showed lower expression in single-dosed vehicle mice as compared with wild type, a trend that was clearer in *Nrf2*-knockout mice.

**TABLE 2:** Fold induction of known NRF2 target gene transcripts in liver of wild-type, *Keap1* C151S mutant, and *Nrf2*-knockout (KO) mice following one or three doses of vehicle or 30 µmol/kg CDDO-Me

		*Nqo1*		*Gstm1*		*Gpx2*		*Srnx1*		*Cbr3*	
		1 Dose	3 Doses	1 Dose	3 Doses	1 Dose	3 Doses	1 Dose	3 Doses	1 Dose	3 Doses
Wild-type	Vehicle	1 ± 0.48*^a^*	1 ± 0.36	1 ± 0.15	1 ± 0.41	1 ± 0.26	1 ± 0.24	1 ± 0.14	1 ± 0.37	1 ± 0.10	1 ± 0.47
	CDDO-Me	7.93 ± 1.25*^b^*	7.06 ± 1.20*^b^*	3.49 ± 1.23*^b^*	3.25 ± 0.67*^b^*	4.54 ± 0.64*^b^*	13.09 ± 3.71*^b^*	4.59 ± 1.37*^b^*	3.04 ± 0.79*^b^*	43.70 ± 13.72*^b^*	69.50 ± 30.57*^b^*
C151S	Vehicle	0.56 ± 0.05	0.68 ± 0.43	0.42 ± 0.20	1.1 ± 1.10	0.59 ± 0.08	0.99 ± 0.50	0.32 ± 0.08	1.05 ± 0.88	0.53 ± 0.16	1.05 ± 0.99
	CDDO-Me	0.62 ± 0.11	0.55 ± 0.44	0.31 ± 0.11	0.42 ± 0.20	0.60 ± 0.17	0.60 ± 0.22	0.41 ± 0.20	1.14 ± 0.31	0.60 ± 0.10	0.70 ± 0.13
*NRF2*KO	Vehicle	0.07 ± 0.03	0.11 ± 0.02	0.07 ± 0.02	0.16 ± 0.02	0.46 ± 0.16	0.38 ± 0.09	0.17 ± 0.02	0.31 ± 0.11	0.18 ± 0.04	0.30 ± 0.08
	CDDO-Me	0.14 ± 0.07	0.11 ± 0.04	0.05 ± 0.01	0.11 ± 0.42	0.42 ± 0.04	0.39 ± 0.06	0.37 ± 0.18	0.26 ± 0.09	0.33 ± 0.19	0.52 ± 0.09*^b^*

*^a^*All values for gene expression levels are shown as mean ± S.D. (N = 3).

^b^*P* < 0.05 using *t* test. After controlling for 30 multiple comparisons with Bonferroni correction, only *Nqo1* (one and three doses) reached statistical significance (*P* < 0.00167).

To investigate whether a point mutation on C151 of KEAP1 affects NRF2 signature pathways with respect to CDDO-Me, the top four pathways found in the Kyoto Encyclopedia of Genes and Genomes database were directly compared between genotypes based on gene set enrichment analysis. Classifications of “metabolism of xenobiotics by cytochrome P450”, “drug meta-bolism – cytochrome P450”, “glutathione metabolism”, “chemical carcinogenesis – DNA adduct” were enriched in CDDO-Me–treated wild-type mice as compared with their counterpart in C151S, which, in turn, was exceedingly similar to the profile between wild type and *Nrf2*-knockout (Fig. 5E), indicating that KEAP1 C151 has primary, if not sole, responsibility in the NRF2 pathways induced by CDDO-Me. In summary, our global transcriptomic analysis demonstrated that a point mutation at KEAP1 C151 has a profound impact on NRF2-dependent gene expression induced by CDDO-Me in mouse liver.

## Discussion

Multiple transcription factors, including NRF2, NF-*κ*B, FOXO, and p53, have prominent amino acids that are reactive toward oxidants and electrophiles to drive stress-related adaptive signaling reactions (Sies et al., 2022). Cysteine thiolate residues are often the centerpieces for these regulatory hubs. Remarkably, it is estimated that 10%–20% of the full complement of 214,000 thiols in the cellular cysteine proteome are readily oxidized under aerobic conditions (Jones, 2008). Far beyond transcription factors, this modifiable proteome includes enzymes, transporters, receptors, cytoskeletal elements, heat shock proteins, and scaffold proteins. Understanding the factors distilling this pleiotropy of reactive targets into finely tuned cellular responses is a matter of current research (Wible and Sutter, 2017; Sies and Jones, 2020; Sies et al., 2022). As discussed in *Introduction*, the NRF2-KEAP1 system is the pioneer paradigm for a physiologic thiol-based sensor-effector apparatus responding to cellular and toxic stresses and provides opportunities to probe the extent of target specificity leading to network responses and their roles in redox medicine.

There are hundreds of small molecules—natural products and synthetic compounds—that activate NRF2 signaling. Most are effective but not especially potent as they elicit responses at micromolar concentrations in cells. These agents likely interact with many accessible thiols in the proteome. Newer classes of inducers such as the oleanane triterpenoids are exceedingly potent (low nanomolar to high picomolar) and may touch few thiol targets at these bioeffective levels. However, the extent to which any of these electrophilic or oxidizing inducers rely on the NRF2 pathway exclusively for their cytoprotective actions is not clear. Nonetheless, there are several examples where the protective effects of triterpenoids administered to mice prior to toxicologic or carcinogenic challenges are profound in wild-type mice, phenocopied in *Keap1-*knockdown mice, and abrogated in *Nrf2*-knockout mice. Such findings indicate that the NRF2 pathway is sufficient for mediating adaptive responses to stress but do not rule out roles for other mechanisms in protection. Indeed, Liby et al. (2007) have posited that triterpenoids are not monofunctional drugs that uniquely target single steps in signal transduction pathways, as reflected by their profound effects on inflammation and the redox state of cells, as well as being potent antiproliferative and proapoptotic agents. Furthering the view of synthetic oleanane triterpenoids as multifunctional drugs targeting multiple disease-relevant signaling networks, the study of Yore et al. (2011) with treatment of HEK293 cells with 4 µM biotinylated triterpenoid followed by affinity purification and mass spectroscopic proteomic analysis led to the identification of 577 candidate binding proteins. Are all of these targets required for efficacy?

Results from our study indicate that activation of NRF2 signaling is a *necessary and sufficient* condition for cytoprotection in cell culture and an immune hepatitis model in mice by simple mutation of a key electrophilic target facilitating action of NRF2: cysteine 151 in the pathway repressor KEAP1. Validation of the model is shown by robust induction of the prototypical NRF2 target gene *Nqo1* in mutant C151S MEFs by non-Cys151 requiring inducers (e.g., 15Δ-PGJ_2_, CdCl_2_) but complete abrogation of response to 30 nM CDDO-Me. No nuclear translocation of Nrf2 was observed in the liver of C151S mice, unlike wild type, following treatment with CDDO-Me. No induction of *Nqo1* transcripts were observed with single or multiple doses in the mutant mice. RNA-seq analysis unequivocally demonstrated that CDDO-Me, administered as a single dose, induced a robust response in hepatic genes of the NRF2 pathway but did not occur in *Nrf2*-knockout mice nor in C151S mutant mice. DEGs reflecting other signaling pathway responses were not detected in wild-type, *Nrf2*-knockout, or C151S mutant mice. Thus, at the dose used, CDDO-Me induced only an NRF2-dependent response. The relevance of this dose is highlighted by the protection against ConA-induced immune hepatitis in wild-type but not C151S mutant mice as adjudged by serum ALT levels. Earlier studies had demonstrated that the genetic homolog (pharmaco-mimetic) to this intervention, *Keap1*-knockdown mice, profoundly protected against ConA hepatitis (Osburn et al., 2008). In both studies, exacerbation of toxicity was not observed in the C151S mutant and *Nrf2*-knockout mice, likely reflecting the selection of a challenge dose optimized to provide a dynamic range to detect protection against rather than enhanced toxicity.

While CDDO-Me is in late stages of clinical development (e.g., the phase 2 and 3 trials: BEAM, NCT00811889; BEACON, NCT01351675; CARDINAL, NCT03019185; PHOENIX, NCT03366337; TSUBAKI, NCT02316821; and AYAME, NCT03550443), we also used CDDO-Im as a comparator in many of our experiments. CDDO-Im has been widely used in rodent studies because of perceived advantages in potency and lower toxicity to mice. No evidence of hepatic toxicity with either single or multiple doses of CDDO-Im or CDDO-Me were observed in our studies as adjudged by serum ALT measures. However, Kamel et al. (2019) has described the metabolism of CDDO-Me to a reactive epoxide in mice that could contribute to toxicity. In the cell culture assays using MEFs, where dose-response relationships can be easily defined, it is clear that CDDO-Me and CDDO-Im are equi-effective and equi-potent at inducing *Nqo1* transcripts in wild-type cells. CDDO-Me elicits no induction at any concentration tested in the C151S mutant cells, whereas CDDO-Im elicits an attenuated response at higher concentrations (e.g., 30 nM). CDDO-Me is C151 requiring, whereas CDDO-Im is C151 preferring. Meng et al. (2020) provide a possible explanation for these divergent responses. CDDO-Me is a monofunctional molecule in which only the A ring of oleanolic acid has been activated. However, the imidazolide moiety attached to the C-28 of CDDO-Im renders it a bifunctional molecule with much greater potential for acylation of nucleophilic amino acid residues. Their proteomic and modeling studies indicate that interaction with Tyr85 in KEAP1 by CDDO-Im, but not CDDO-Me, stabilizes the interaction with KEAP1 to potentially enhance potency and/or durability of the interaction.

Our study with CDDO-Me and KEAP1 C151S provides the second example of the role of specific cysteine amino acids as determinates of the NRF2 activation in vivo. Suzuki et al. (2019) developed stabile11-cysteine-less MEF lines that were unable to respond to cysteine-reactive NRF2 inducers, including CDDO-Im, 15Δ-PGJ_2_, and CdCl_2_. Four of those cysteines (Cys226/613/622/624) were important for sensing hydrogen peroxide. Mechanistic dissection using a series of mutant MEFs and mice indicated that Cys226, Cys613, and Cys622/Cys624 form an elaborate fail-safe mechanism in which all of these cysteine residues contribute to hydrogen peroxide sensing by KEAP1, but no single residue is essential for a response to oxidative stress in vivo. Thus, hydrogen peroxide and CDDO-Im, unlike CDDO-Me, appear to use multiple amino acid interactions to evoke NRF2 activation.

Many NRF2 inducers have been characterized as KEAP1 C151–preferring based upon analyses in cysteine-mutant MEFs, including the triterpenoids, dimethyl fumarate (DMF), and sulforaphane. To et al. (2015) observed that CDDO-Im and CDDO-Me were markedly more potent than DMF for activation of the NRF2 pathway in RAW 264.7 mouse macrophage-like cells. However, microarray analysis indicated that only half (52 of 99) of the NRF2 target genes were induced by all three; further, each drug regulated a unique subset of NRF2 genes. Moreover, effects in the A/J model of lung carcinogenesis were different: CDDO-Im and CDDO-Me showed protective effects, whereas DMF elevated sensitivity to lung carcinogenesis in this model. Similarly, Wible et al. (2018) used a chemical genomics approach to characterize the hepatic expression profiles between CDDO-Im and 1,2-dithiole-3-thione (D3T) from wild-type and *Nrf2*-knockout mice. At equally efficacious doses in wild-type mice, 406 genes enriched in the NRF2-regulated pathways of antioxidant defense and xenobiotic metabolism showed common RNA responses to both treatments. However, CDDO-Im resulted in a unilateral, NRF2-dependent elevation of peroxisome proliferator *α* and Kruppel-like factor 13, as well as the peroxisome proliferator *γ* coactivator 1b, together indicating regulation of *β*-oxidation and lipid metabolic pathways. By contrast, functional analysis of the D3T-regulated set showed a robust enrichment of NRF2-regulated enzymes involved in cholesterol biosynthesis wherein CDDO-Im had no effect on cholesterol biosynthesis. The molecular determinants to these unique, NRF2-dependent responses of the two inducers are unclear at present.

Our study highlights the efficacy in vitro and in vivo of a potent, monofunctional molecule that selectively activates the NRF2 stress response pathway in a mechanism that is solely dependent upon interaction with a single cysteine amino acid, C151, in KEAP1. However, more studies will be required to determine the optimal features of triterpenoid action in the development of best-in-class triterpenoids for prophylactic and therapeutic uses. Are monofunctional or bifunctional molecules preferred? CDDO-Me versus CDDO-Im comparisons are ambiguous at present, with some studies showing greater or lower efficacy depending upon the cell system or animal model used. Is CDDO-Me the “best” triterpenoid? Features of pathway specificity versus broad network responses need careful consideration. What defines target versus off-target interactions? How does dose or metabolism influence the considerations of pathway specificity or toxicity? At a minimum, CDDO-Me is a useful pharmacological probe for potent induction of NRF2 signaling. At best, it will prove to be a selective, safe, and highly efficacious agent for disease prevention or amelioration. Or, as others suggest, if chemical and pathway multifunctionality is optimal, then new molecules that include better bioavailability, metabolic stability, and accessibility to all compartments, including the brain, will need to be developed.

## Abbreviations

ALT: alanine aminotransferase
ARE: antioxidant response element
BW: body weight
C151: cysteine 151
CBR3: carbonyl reductase 3
CDDO-Im: 1-(2-cyano-3,12,28-trioxooleana-1,9(11)-dien-28-yl)-1H-imidazole
CDDO-Me: methyl-2-cyano-3,12-dioxooleano-1,9-dien-28-oate (bardoxolone methyl)
ConA: concanavalin A
D3T: 1,2-dithiole-3-thione
DEGs: differentially expressed genes
DMF: dimethyl fumarate
FC: fold change
FDR: false discovery rate
GSTM1: glutathione *S*-transferase M1
KEAP1: kelch-like ECH-associated protein 1
MEFs: mouse embryo fibroblasts
Menadione: 2-methyl-1,4-naphthoquinone
MTT: thiazolyl blue tetrazolium bromide
NQO1: NAD(P)H:quinone dehydrogenase 1
NRF2: NF-E2-related factor 2
qPCR: quantitative real-time polymerase chain reaction
